# Uncovering the biotechnological capacity of marine and brackish water *Planctomycetota*

**DOI:** 10.1007/s10482-023-01923-z

**Published:** 2024-01-23

**Authors:** Inês R. Vitorino, Eugénia Pinto, Jesús Martín, Thomas A. Mackenzie, Maria C. Ramos, Pilar Sánchez, Mercedes de la Cruz, Francisca Vicente, Vítor Vasconcelos, Fernando Reyes, Olga M. Lage

**Affiliations:** 1 Department of Biology, Faculty of Sciences, University of Porto, Rua Do Campo Alegre S/N, 4169-007 Porto, Portugal; 2 CIIMAR/CIMAR, Interdisciplinary Centre of Marine and Environmental Research, Terminal de Cruzeiros Do Porto de Leixões, 4450-208 Matosinhos, Portugal; 3 Laboratory of Microbiology, Biological Sciences Department, Faculty of Pharmacy, University of Porto, 4050-313 Porto, Portugal; 4 Fundación MEDINA, PTS Health Sciences Technology Park, Avenida del Conocimiento 34, 18016 Granada, Spain

**Keywords:** *Planctomycetia*, Oligotrophy, Antibacterial, Antifungal, Anti-tumour, LC-HRMS

## Abstract

An appealing strategy for finding novel bioactive molecules in Nature consists in exploring underrepresented and -studied microorganisms. Here, we investigated the antimicrobial and tumoral anti-proliferative bioactivities of twenty-three marine and estuarine bacteria of the fascinating phylum *Planctomycetota*. This was achieved through extraction of compounds produced by the *Planctomycetota* cultured in oligotrophic medium followed by an antimicrobial screening against ten relevant human pathogens including Gram-positive and Gram-negative bacteria, and fungi. Cytotoxic effects of the extracts were also evaluated against five tumoral cell lines. Moderate to potent activities were obtained against *Enterococcus faecalis,* methicillin-sensitive and methicillin-resistant *Staphylococcus aureus* and vancomycin-sensitive and vancomycin-resistant *Enterococcus faecium*. Anti-fungal effects were observed against *Trichophyton rubrum, Candida albicans* and *Aspergillus fumigatus*. The highest cytotoxic effects were observed against human breast, pancreas and melanoma tumoral cell lines. *Novipirellula caenicola* and *Rhodopirellula* spp. strains displayed the widest spectrum of bioactivities while *Rubinisphaera margarita* ICM_H10^T ^affected all Gram-positive bacteria tested. LC-HRMS analysis of the extracts did not reveal the presence of any known bioactive natural product, suggesting that the observed activities are most likely caused by novel molecules, that need identification. In summary, we expanded the scope of planctomycetal species investigated for bioactivities and demonstrated that various strains are promising sources of novel bioactive compounds, which reenforces the potential biotechnological prospects offered by *Planctomycetota*.

**Supplementary Information:**

The online version contains supplementary material available at 10.1007/s10482-023-01923-z.

## Introduction

Humanity is currently facing a shortage of effective drugs for many of its health challenges. Noncommunicable diseases, which include cancer, diabetes, obesity, cardiovascular diseases and chronic respiratory conditions, are presently accountable for more than 70% of the world deaths (WHO 2019a). Particularly, cancer alone was responsible for around 10 million worldwide deaths in 2020. By this date, lung and colon cancer were the most deadly, while breast cancer was the most common (WHO 2019a). Although prevention and early detections are crucial in reducing the burden of cancer, finding novel effective treatments with lower side effects is also of great importance for improving the recovery rates and the quality of life of patients undergoing therapy. On another perspective, perhaps one of the most worrying health problems today is the continuous rise of antibiotic resistance in microbial pathogens (WHO 2017, 2019b). According to the World Health Organization (WHO), deaths caused by resistant microorganisms have the tendency to increase exponentially. Bacteria classified as critical and high priority by WHO include antibiotic resistant *Pseudomonas aeruginosa, Acinetobacter baumannii*, *Neisseria gonorrhoeae*, *Staphylococcus aureus*, *Helicobacter pylori*, *Campylobacter* sp., *Salmonella* sp. and many *Enterobacteriaceae* (WHO 2017), as well as various fungi (WHO 2022). The still ongoing COVID-19 outbreak revealed how complex a worldwide epidemy can be regarding public health and socioeconomic impact. In the verge of the imminent possibility of new pandemics caused by resistant microorganisms, we are now in need of novel successful medications.

Nature has always been a shear unlimited source for bioactive molecules and, in particular, still untapped environments such as the Oceans have gained attention in recent years due to their rich biological and metabolic diversities (Gerwick and Moore 2012; Santos, et al. 2020b). In particular, microorganisms offer many advantages such as ease of manipulation and large potential for the production of many bioactive compounds and promising leads. In fact, many known antimicrobials come from (marine) microorganisms (Santos, et al. 2020b). Marine *Actinomycetota*, *Pseudomonadota, Bacillota, Bacteroidota*, *Cyanobacteria*, fungi and dinoflagellates have shown to be excellent sources of metabolite diversity (Anjum, et al. 2018; Braña, et al. 2017; Feling, et al. 2003; Harinantenaina Rakotondraibe, et al. 2015; Leao, et al. 2013; Reynolds, et al. 2018; Rodriguez, et al. 2018; Schulze, et al. 2015; Tareq, et al. 2014; Wiese, et al. 2018; Zhang, et al. 2018, 2016). Nevertheless, they have been more extensively studied in the past decades, while other microbial groups still remain underexplored. An attractive approach for finding novel effective drugs and lower rediscovery rates would be to investigate these underexplored microorganisms.

The vast majority of estimated bacterial diversity is still uncultured in laboratory (Barer and Harwood 1999; Rinke, et al. 2013), which heavily restricts what could be screened for biotechnological purposes. Nonetheless, recent cultivation efforts from diverse research groups have allowed to bring into axenic culture many strains (including novel species) from underexplored and evolutionarily deep-branching groups such as the *Planctomycetota* (Almeida, et al. 2022, Dedysh, et al. 2020, Gaurav, et al. 2021, Kaushik, et al. 2020, Kulichevskaya, et al. 2020a, Kulichevskaya, et al. 2022, Kulichevskaya, et al. 2020b, Kumar, et al. 2020a, Kumar, et al. 2020b, Kumar, et al. 2021a, 2021b, Vitorino, et al. 2020, Vitorino et al. 2021b, Vitorino, et al. 2022b, Vitorino, et al. 2022c, Vitorino, et al. 2022d, Wiegand, et al. 2020). The bacterial phylum *Planctomycetes* (now renamed *Planctomycetota* (Oren and Garrity 2021)) belongs to the super phylum *Planctomycetota*-*Chlamydiota*-*Verrucomicrobiota* (PVC) (Rivas-Marín and Devos 2018; Wagner and Horn 2006). *Planctomycetota* have many interesting and distinctive features that makes them relevant bacteria from an ecological and a biological point of view (Lage, et al. 2019; Wiegand, et al. 2018). They can inhabit most of the earth’s biomes and play central roles in the carbon and nitrogen cycles (Lage and Bondoso 2014; Lage, et al. 2019). Their cells have permanently condensed DNA, unusual structures and complex cellular plans with intricate invaginations of the cytoplasmatic membrane, large periplasmic space and an enigmatic cell wall (Boedeker, et al. 2017; Devos 2014; Jeske, et al. 2015; Lage, et al. 2013; Santarella-Mellwig, et al. 2013). *Planctomycetota* cell division can be by binary fission (*Phycisphaerae* and *Candidatus* “Brocadiia”) (Fukunaga, et al. 2009; Jetten, et al. 2010; Lodha, et al. 2021) or by a rare prokaryotic budding (*Planctomycetia*) (Krieg et al. 2010; Vitorino and Lage 2022). This division is performed without the otherwise universal FtsZ protein, and the exact process is still unknown (Rivas-Marin, et al. 2020). Endocytosis like uptake of molecules and membranous tubulovesicular networks were also proposed for these bacteria (Acehan, et al. 2014; Boedeker, et al. 2017).

It was only in the recent years that *Planctomycetota* have gained attention as promising organisms for biotechnological purposes due to the lack of isolates (< 200) (Kallscheuer and Jogler 2021; Vitorino and Lage 2022). Furthermore, *Planctomycetota* do not grow as fast or are not as easy to handle as other microorganisms, so they often require complex media formulations and long cultivation periods. Even so, insights into the biology of these bacteria have highlighted their potential as producers of molecules of pharmacological interest. Most *Planctomycetota* have large genomes with many genes of unknown function and a comparatively high number of genes coding large proteins (Faria, et al. 2018; Lage and Bondoso 2014; Wiegand, et al. 2018), features that are shared with other well-known producers of bioactive compounds. Genome mining in many strains also demonstrated their rich content of putatively new biosynthetic gene clusters (BGCs) from several structural types (such as nonribosomal peptide synthases (NRPS) polyketide synthases (PKS), ribosomally synthesized and post-translationally modified peptides (RiPPs), lanthipeptides and bacteriocins), which are often linked to the production of bioactive secondary metabolites (Belova, et al. 2020; Graca, et al. 2016; Jeske, et al. 2013; Kallscheuer and Jogler 2021; Vitorino, et al. 2022b, 2022d; Wiegand, et al. 2020). Additionally, the capability of some *Planctomycetota* to produce antimicrobial and cytotoxic molecules was already confirmed through in vitro bioactivity screenings, including the description of the first (and the only one up to date) antimicrobial compounds from planctomycetal origin (Belova, et al. 2020; Calisto, et al. 2019; Graca, et al. 2016; Jeske, et al. 2016; Kallscheuer, et al. 2020; Sandargo, et al. 2020). These N-acyl-amino acid molecules were named Stieleriacines A-E and were isolated from two species in the genus *Stieleria* (Kallscheuer, et al. 2020; Sandargo, et al. 2020). Although these studies begin to show the great potential for biosynthesis of natural products in the *Planctomycetota*, the spectrum of planctomycetal strains and species studied remains limited.

**Fig. 1:**
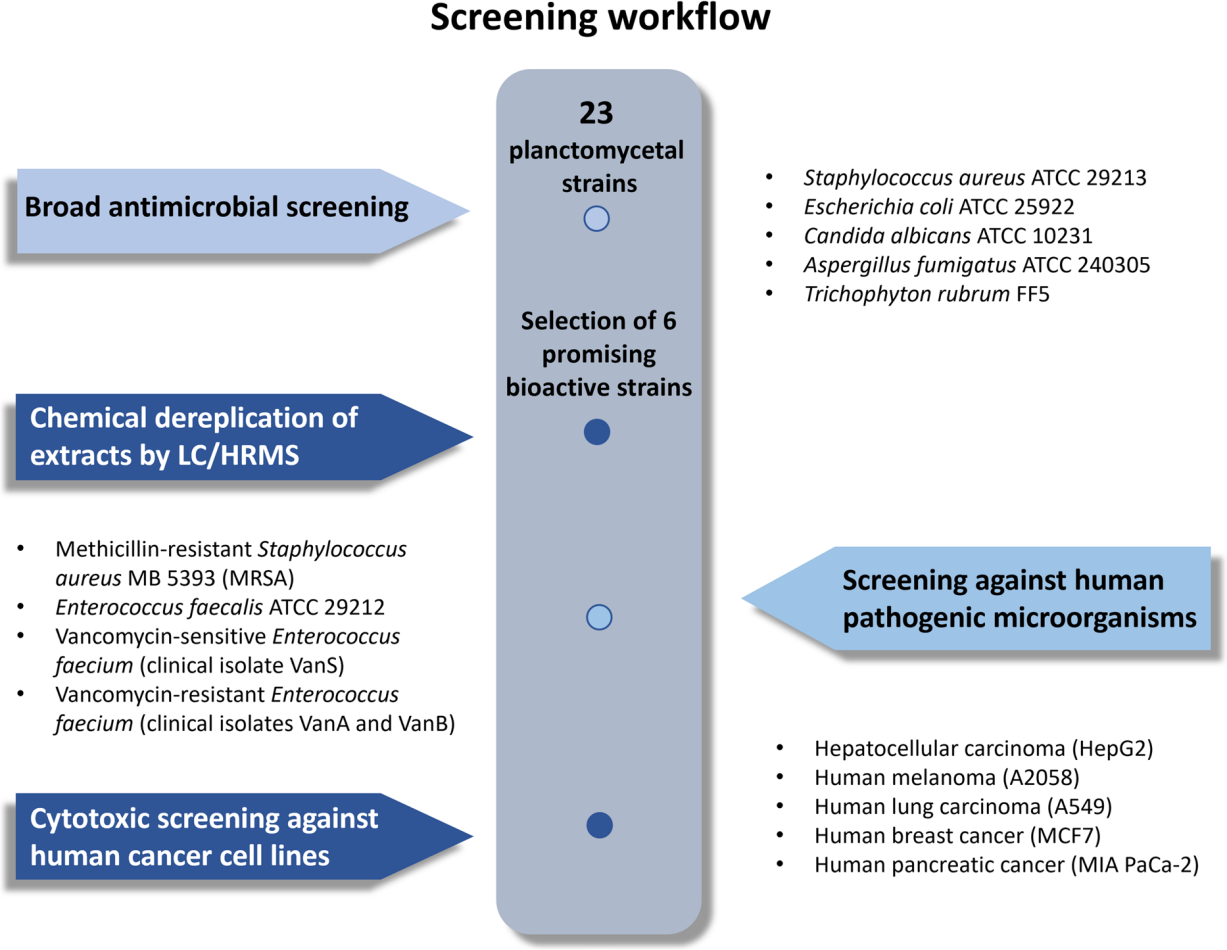
Schematic representation of the screening workflow followed in this study

The demand for new chemically distinct molecules, combined with the potential of the promising phylum *Planctomycetota*, motivated us to study twenty-three marine and brackish water planctomycetal strains from our culture collection. They were screened for various bioactivities following the workflow represented in Fig. 1. Novel recently described species (Godinho et al. 2021; Vitorino, et al. 2020, 2021a, 2022a, 2022b, 2022c, 2023) were also included in this study. Metabolites from *Planctomycetota* cultured in oligotrophic medium were extracted with organic solvents and followed by antimicrobial and antifungal assays, in a first stage, against two different types of bacteria (the Gram-negative *Escherichia coli* and the Gram-positive *Staphylococcus aureus*) and three different fungi (the yeast *Candida albicans* and the filamentous fungi *Trichophyton rubrum* and *Aspergillus fumigatus*). In the second stage, extracts obtained from the selected bioactive strains were additionally screened against five human pathogens (drug resistant and drug sensitive Gram-positive bacteria) and against five human tumoral cell lines (Fig. 1). Planctomycetal crude extracts were also analyzed by liquid chromatography coupled to high-resolution mass spectrometry (LC-HRMS), which allowed the prediction of molecular formulae of the components present in these extracts and their search against natural products databases.

## Material and Methods

### Biological material

The biological material analyzed in this study included twenty-three planctomycetal strains belonging to our culture collection (Laboratory for Microbial Ecophysiology of the University of Porto, Portugal-LEMUP). These strains were previously isolated from different samples collected in marine and estuarine environments in various regions of Portugal (Bondoso, et al. 2014, 2015; Godinho et al. 2021; Lage and Bondoso 2011; Vitorino, et al. 2020, 2021b, 2022b, 2022c, 2023, 2022d). They are all affiliated to the class *Planctomycetia* within the phylum *Planctomycetota*. The diversity screened in this study includes *Planctomycetota* from three different families (*Pirellulaceae*/*Lacipirellulaceae* and *Planctomycetaceae*) of orders *Pirellulales* and *Planctomycetales* in a total of nine genera and thirteen different species, including recently described novel taxa (Vitorino, et al. 2020, 2021b, 2022c, 2023, 2022d).

All 16S rRNA gene sequences are deposited in the National Center for Biotechnology Information (NCBI) and the respective GenBank accession numbers presented in Table 1, where a summary of the isolation habitat and taxonomic features of each strain are also specified. Maximum likelihood 16S rRNA gene sequence-based phylogenetic trees were constructed to show their position in the phylum together with the results obtained. Sequences were first aligned with Clustal W (Larkin, et al. 2007) together with publicly sequences from the closest type strains, which were retrieved from NCBI under the following GenBank tags: LR132072 for *Gimesia chilikensis* JC646^T^, MK554521 for *Alienimonas californiensis* CA12^T^, AJ231190 for *Rubinisphaera brasiliensis* IFAM 1448^ T^, MK559971 for *Stieleria neptunia* Enr13^T^, MK554549 for *Novipirellula galeiformis* Pla52a^T^, NR_136448 for *Novipirellula caenicola* YM26-125^ T^, NR_043384 for *Rhodopirellula baltica* SH1^T^, EF589351 for *Rhodopirellula lusitana* UC17^T^, AP021861 for *Lacipirellula parvula* PX69^T^, KF607112 for *Bythopirellula goksoeyrii* Pr1d^T^, MK559982 for *Aeoliella mucimassa* Pan181^T^. The trees were constructed using the Mega X software with 1000 bootstraps replicates following the general time reversible model and activated gamma distributed with invariant sites (G + I) (Kumar, et al. 2018). iTOL was used for tree visualization and annotation (Letunic and Bork 2021).

**Table 1:** Summary of the main features of the planctomycetal strains explored in this study

Strain ID	Affiliation (species/ family)	Origin (in Portugal)	Isolation source	*GenBank accession number
Pd1	*Gimesia chilikensis / Planctomycetaceae*	Beach in Mindelo^a^	Macroalgae	HQ845497
LzC1	*Alienimonas chondri/ Planctomycetaceae*	Beach in Porto^b^	Macroalgae	MW669960
LzC2^T^	*Alienimonas chondri/ Planctomycetaceae*	Beach in Porto^b,c^	Macroalgae	MN757873
ICM_H10^T^	*Rubinisphaera margarita/ Planctomycetaceae*	Beach in Matosinhos^b,d^	Sediments	MW588631
Gr7	*Rubinisphaera brasiliensis/ Planctomycetaceae*	Aveiro^a^	Macroalgae	HQ845477
UC8^T^	*Roseimaritima ulvae/ Pirellulaceae*	Beach in Carreço^a,e^	Macroalgae	NR_146676
ICT_E10.1^ T^	*Stieleria sedimenti/ Pirellulaceae*	Tagus river estuary^f,h^	Sediments	OL684514
ICM_H5	*Novipirellula caenicola/ Pirellulaceae*	Beach in Matosinhos^b^	Sediments	MW669970
ICM_G4	*Novipirellula caenicola/ Pirellulaceae*	Beach in Matosinhos^b^	Sediments	MW669968
LzA1	*Novipirellula caenicola/ Pirellulaceae*	Beach in Porto^b^	Seawater	MW669961
MEMO17_8	*Novipirellula caenicola/ Pirellulaceae*	Beach in Matosinhos^b^	Macroalgae	MW669969
MTI8c	*Rhodopirellula baltica/ Pirellulaceae*	Tagus river estuary^f^	Macroalgae	OL684473
MsF2	*Rhodopirellula baltica/ Pirellulaceae*	Beach in Porto^a^	Macroalgae	HQ845493
ICT_H6.1	*Rhodopirellula baltica/ Pirellulaceae*	Tagus river estuary^f^	Sediments	OL684515
MEMO26_1	*Rhodopirellula baltica/ Pirellulaceae*	Beach in Matosinhos^b^	Macroalgae	MW669971
LzF4	*Rhodopirellula lusitana/ Pirellulaceae*	Beach in Porto^b^	Macroalgae	MW669966
ICT_H3.1^ T^	*Rhodopirellula aestuarii/ Pirellulaceae*	Tagus river estuary^f,i^	Sediments	OK001858
ICT_E8.1	*Rhodopirellula rubra/ Pirellulaceae*	Tagus river estuary^f^	Sediments	OL684489
LF2^T^	*Rhodopirellula rubra/ Pirellulaceae*	Beach in Porto^a,g^	Macroalgae	NR_126223
MTI7a	*Rhodopirellula rubra/ Pirellulaceae*	Tagus river estuary^f^	Macroalgae	OL684474
FF15^T^	*Bremerella alba/ Pirellulaceae*	Beach in Porto^a,j^	Macroalgae	HQ845465
ICT_H6.2^ T^	*Aeoliella* straminimaris* / Lacipirellulaceae*	Tagus river estuary^k^	Sediments	OM714897
ICT_H3.7	*Aeoliella* straminimaris* /Lacipirellulaceae*	Tagus river estuary	Sediments	OP474150

^a^Lage and Bondoso (2011), ^b^Vitorino*, *et al*.* (2021b), ^c^Vitorino, et al. (2020), ^d^Vitorino, et al. (2022c), ^e^Bondoso, et al. (2015), ^f^Vitorino, et al. (2022d), ^g^Bondoso, et al. (2014), ^h^Vitorino, et al. (2022b), ^i^Vitorino, et al. (2022a), ^j^Godinho, et al. (2021), ^k^Vitorino, et al. (2023)

*Partial 16S rRNA gene sequence

### Culturing of strains and natural products extraction

Strains were routinely maintained in pure culture at 25 °C in modified M13 medium plates prepared as previously described (Lage and Bondoso 2011). For metabolite extraction, strains were first cultured at 25 °C for 7 days in a liquid pre-inoculum in modified M13 medium prepared as follow: per liter of medium, 920 mL of deionized water, 0.25 g of yeast extract and peptone, 50 mL of HCl-Tris buffer (0.1 mM, pH 7.5) and 33 g of sea salts (Sigma-Aldrich®, St. Louis, MO, USA). After autoclaving, the following solutions were added by sterile filtration (0.22 µm): 10 mL glucose solution (2.5% w/v), 10 mL vitamin solution (following previous formulations (Lage and Bondoso 2011)) and 20 mL Hutner’s basal salts (prepared as described formerly (Cohen-Bazire, et al. 1957). To enhance secondary metabolite production, nutritional stress was induced. The pre-inoculums were diluted 1:5 in 250 mL of the oligotrophic 1:10 M13 medium (Vitorino, et al. 2021b), prepared as follows, per liter of medium: 919 mL of deionized water, 0.025 g of yeast extract and peptone, 50 mL of HCl-Tris buffer (0.1 mM, pH 7.5) and 33 g of sea salts (Sigma-Aldrich®, St. Louis, MO, USA). After autoclaving, the following solutions were added by sterile filtration (0.22 µm): 1 mL glucose solution (2.5% w/v), 10 mL vitamin solution (Lage and Bondoso 2011) and 20 mL Hutner’s basal salts (Cohen-Bazire, et al. 1957). After incubation in glass flasks for 14 days at 25 °C under agitation in the absence of light, cells were harvested by centrifugation (3600 rpm for 15 min in an Eppendorf 5810R Centrifuge) and metabolites extracted using acetone (250 mL) for 1–4 h. Solid residues were achieved by drying the solvent in a rotatory vacuum evaporator (Rotavapor® R-100 equipment from BUCHI). Finally, the crude extracts were suspended in 20% v/v dimethyl sulfoxide (DMSO) in water and stored under refrigerated conditions for subsequent screenings.

### Antibacterial assays

All antibacterial assays were performed as previously described, using liquid cultures in a 96-well plate format (Audoin, et al. 2013; Santos, et al. 2020a; Vitorino, et al. 2022d). The two bacterial targets initially tested in this study were methicillin-sensitive *Staphylococcus aureus* (MSSA) ATCC 29213 and *Escherichia coli* ATCC 25922 (Gram-positive and Gram-negative representatives, respectively). Briefly, the pathogens were cultured overnight (30 °C under 220 rpm) in nutrient broth (NB) medium (5 g of peptone, 1 g of yeast extract, 1 L of deionized water) and the cultures standardized to an assay inoculum of 5.0 × 10^5^ colony forming units (CFU)/mL. In the antibacterial assay, 10 μL of each planctomycetal extract were diluted 1:10 with the bacterial inoculum for a final volume of 100 μL per well (assay concentration of extract: 1 mg/mL). Internal plate controls were included for validation of the assay: blank control (100 μL of NB medium), growth control (90 μL target inoculum + 10 μL Millipore water), positive control (90 μL target inoculum + 10 μL ampicillin 200 mg/L) and solvent control (90 μL target inoculum + 10 μL DMSO 20% v/v). Three biological independent experiments (n = 3) were conducted. Turbidity (absorbance (Abs) at 600 nm) was measured at the beginning (T0) and after 24 h of incubation (Tf) at 30 °C in a Multiskan GO plate reader from Thermo Scientific™ equipment. The inhibition percentages were calculated as previously described (Vitorino, et al. 2022d).

Additionally, promising bioactive planctomycetal strains were also screened for a panel of pathogenic microorganisms from Fundación MEDINA’s collection (Fig. 1). These included methicillin-resistant *Staphylococcus aureus* MB 5393 (MRSA), *Enterococcus faecalis* ATCC 29212, vancomycin-resistant *Enterococcus faecium* (clinical isolates VanA and VanB) and vancomycin-sensitive *Enterococcus faecium* (clinical isolate VanS). Briefly, the bacterial pathogens were incubated overnight at 37 °C under 220 rpm in Bacto™ Brain heart infusion (BHI) medium (Becton, Dickinson and company, MD, USA). For the assay, they were diluted to standardized cultures of 1.1 × 10^6^ CFU/mL for MRSA and 5.0 × 10^5^ CFU/mL for all *Enterococcus* spp*.* strains. In the assay, 90 µL per well of the corresponding diluted inoculum were mixed with 10 µL per well of the corresponding extract. Turbidity (Abs_612_ nm) was measured with an Envision plate reader at the beginning (T0) and after 24 h incubation (Tf). Positive and negative internal plate controls were included following the previously described methodologies (Audoin, et al. 2013; Oluwabusola, et al. 2021) and three biological independent experiments (n = 3) were conducted. The Genedata Screener software (Genedata, Inc., Basel, Switzerland) was used for the analysis of the data obtained and to calculate the percentage of inhibition of each extract. The RZ’ factor was used to estimate the robustness of the assays (Zhang, et al. 1999), which was always between 0.87 and 0.95.

### Antifungal assays

The target fungal strains utilized in this study were *Candida albicans* ATCC 10231, *Aspergillus fumigatus* ATCC 240305 and a clinical strain of *Trichophyton rubrum* (FF5), strains that belong to the Mycological Laboratory from the Faculty of Pharmacy, University of Porto (Portugal). The antifungal assays were performed in liquid format using 96-well plates following protocols described previously (Benoutman, et al. 2022; Erbiai, et al. 2021) and according to the Clinical and Laboratory Standard Institute-CLSI guidelines (M38-A2 for filamentous fungi and M27-A3 for yeasts). Briefly, fungal organisms previously cultured in Sabouraud dextrose agar (SDA) were suspended in RPMI-1640 broth buffered with MOPS (pH 7.0 (Sigma-Aldrich®, St. Louis, MO, USA): 1–3 × 10^3^ CFU/mL for *T. rubrum*, 0.4–5 × 10^4^ CFU/mL for *A. fumigatus* and 1–5 × 10^3^ CFU/mL for *C. albicans*. The planctomycetal extracts were then diluted 1:10 with each fungal suspension at an assay concentration of 0.1 mg/mL for a final volume of 200 μL per well. Internal plate controls were added: positive control (1 µg/mL voriconazole for *A. fumigatus* and 64 µg/mL fluconazole for *T. rubrum* and *C. albicans*), blank control (200 μL of RPMI-1640 medium), negative control (180 μL target inoculum + 20 μL of RPMI-1640 medium) and solvent control (180 μL target inoculum + 20 μL of 20% v/v DMSO). The growth of the fungal targets was visually inspected after 2 days at 37 °C for *A. fumigatus* and *C. albicans* and after 7 days at 26 °C for *T. rubrum*. All screenings were done in three biological independent assays (n = 3).

### Cytotoxicity screening

Cytotoxic activities of planctomycetal extracts against five tumoral human cell lines were evaluated using the MTT test for assessing cell viability (Mosmann 1983) in a 96-well-plate format. The assay workflow was made according to MEDINA’s portfolio of high-throughput screenings (Subko, et al. 2021). The cell lines used were human lung carcinoma A549 (ATCC CCL-185), breast adenocarcinoma MCF7 (ATCC HTB-22), human skin melanoma A2058 (ATCC CRL-11147), hepatocyte carcinoma HepG2 (ATCC HB-8065) and pancreas carcinoma MiaPaca-2 (ATCC CRL-1420). Extracts were tested at 0.25 mg/mL per duplicate for 72 h and the data obtained analysed using Genedata Screener Software.

### Analysis of extracts by liquid chromatography/high-resolution mass-spectrometry

Bioactive extracts were subjected to a chemical dereplication analysis, the process of identification of already known bioactive compounds in an extract (Perez-Victoria, et al. 2016), in this case achieved by combined liquid chromatography/high-resolution mass-spectrometry (LC-HRMS) techniques. The equipment used was an Agilent 1200 Rapid Resolution HPLC interfaced with a Bruker maXis mass spectrometer. The stationary phase utilized was a Zorbax SB-C8 column (2.1 mm × 30 mm, 3.5 mm particle size) and the mobile phase (10-min run) constituted by two solvents, both composed of water (A) and acetonitrile (B), in a 90:10 ratio for solvent A, and in a 10:90 ratio for B, both additionally supplemented with ammonium formate (13 mM) and 0.01% trifluoracetic acid. The mass spectrometer was operated in positive ESI mode. For putative component identification, the retention time and exact mass of each element were compared against Fundación MEDINA’s high-resolution mass spectrometry database. Additionally, the predicted molecular formulae or the exact masses were searched for in the Chapman and Hall Dictionary of Natural Products (DNP) database (https://dnp.chemnetbase.com/chemical/ChemicalSearch.xhtml?dswid=3475, accessed on June 2023).

## Results and Discussion

### Antimicrobial activities of marine and estuarine *Planctomycetota*

**Fig. 2:**
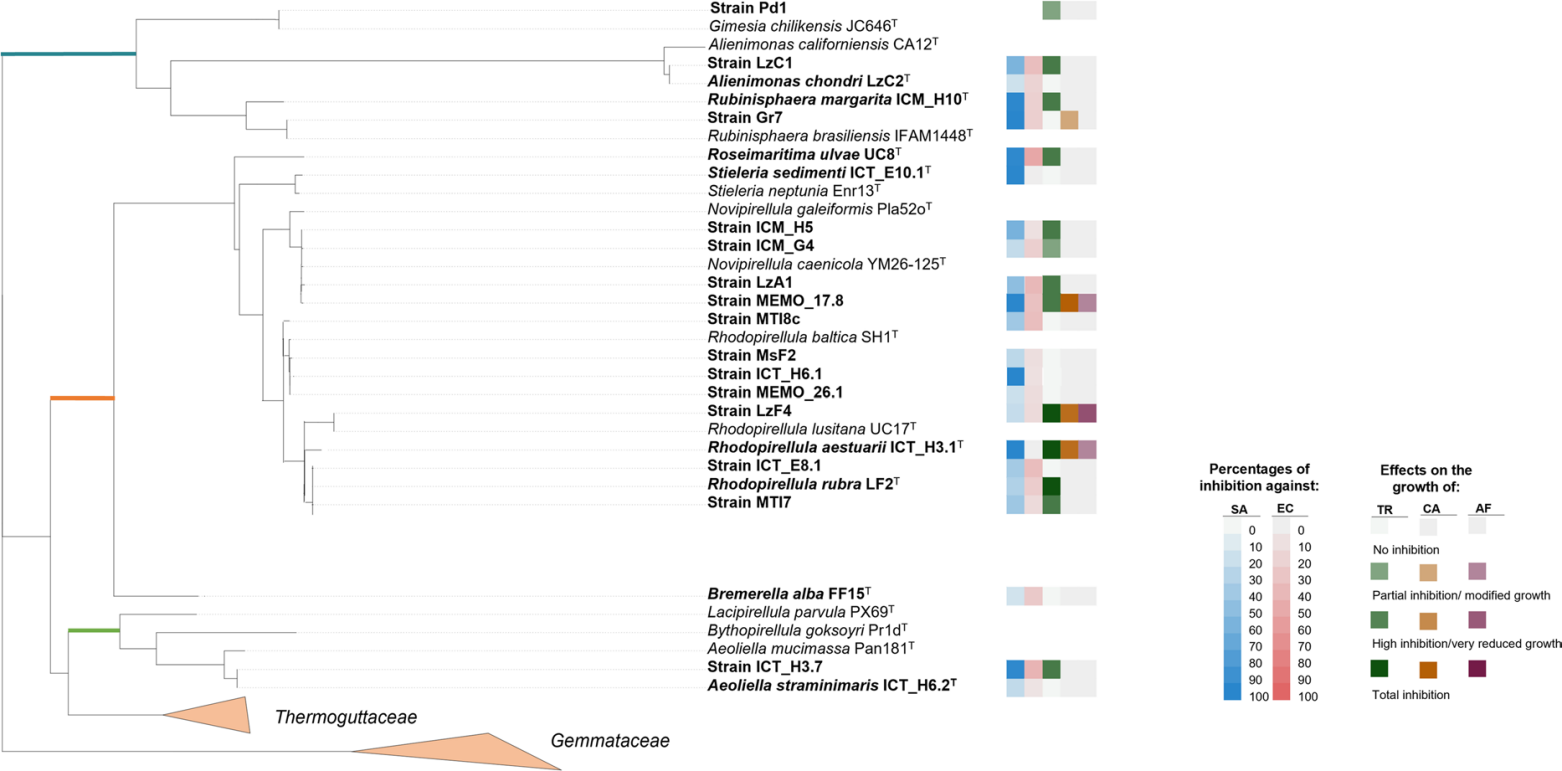
Phylogenetic representation of the planctomycetal strains screened for antimicrobial activities in this study (in bold) together with a heat map showing the effects against each of the following microbial targets: SA = methicillin-sensitive *Staphylococcus aureus* ATCC 29213; EC = *Escherichia coli* ATCC 25922; TR = *Trichophyton rubrum* FF5; CA = *Candida albicans* ATCC 10231 and AF = *Aspergillus fumigatus* ATCC 240305. Data used for construction of this tree is presented in Supplementary Table 1. Coloured branches were used to differentiate each family: green for *Lacipirellulaceae*, orange for *Pirellulaceae* and blue for *Planctomycetaceae*. *data on the antibacterial effects of strain ICT_E10.1^T^ were taken from previous study (Vitorino, et al. 2022b) and represented here for comparison

In total, twenty-three marine and brackish water planctomycetal strains were screened for antimicrobial activities against different microorganisms following the workflow depicted in Fig. 1. The microorganisms included in this study represent both types of Gram-positive and Gram-negative bacteria as well as other human pathogens (including fungi and drug-resistant bacteria) and human tumoral cell lines. A maximum-likelihood tree was constructed to show the phylogenetic position of each planctomycetal strain together with a heat map to summarize the antimicrobial results obtained (Fig. 2). The distribution of these planctomycetal bioactivities across bacterial and fungal pathogens is also displayed in Supplementary Fig. 1.

**Fig. 3:**
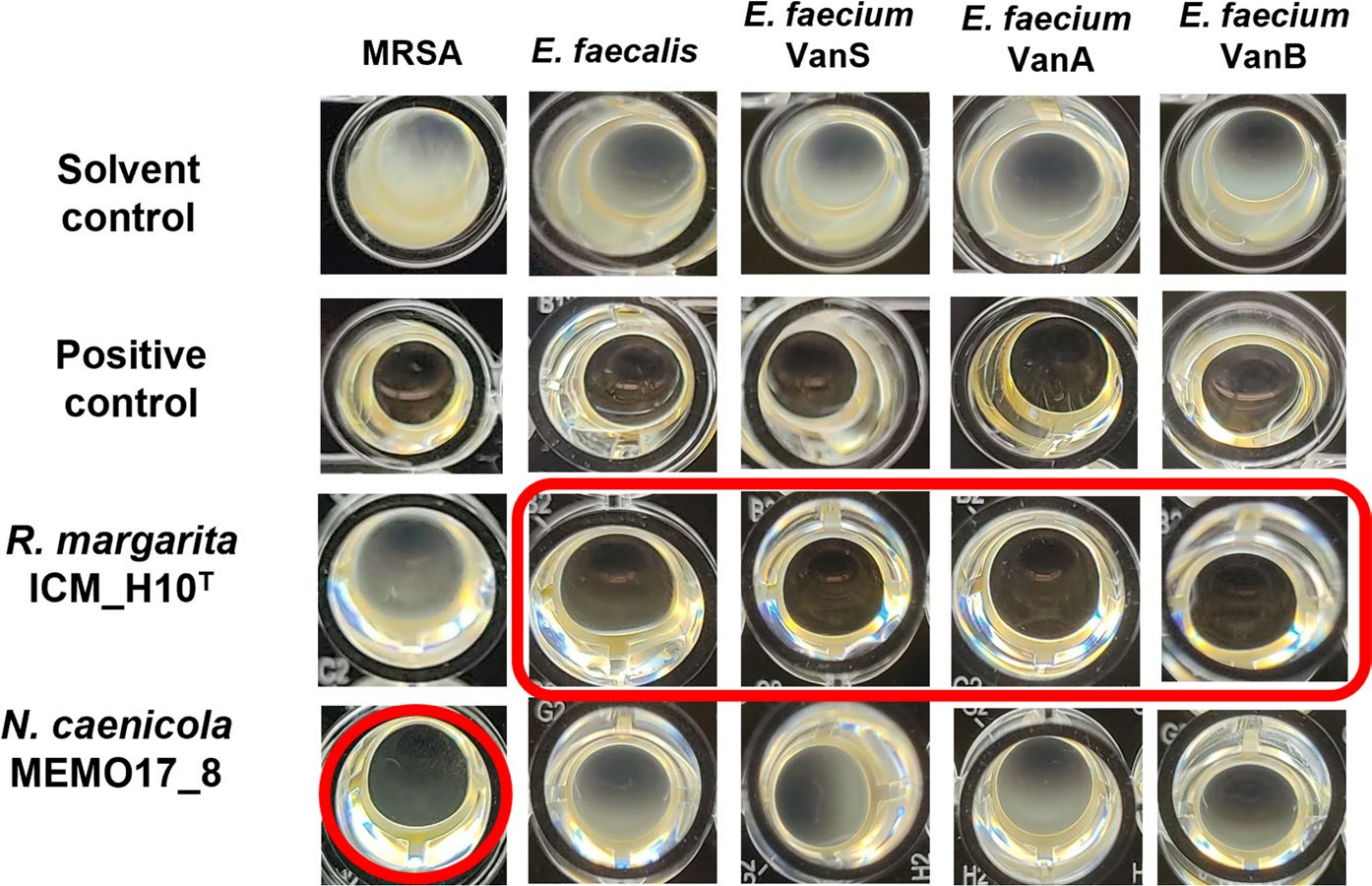
Assay wells of the antimicrobial screening of *Novipirellula caenicola* MEMO17_8 and *Rubinisphaera margarita* ICM_H10^T^ extracts showing the potent inhibitory effects of these strains against methicillin-resistant *Staphylococcus aureus* MB 5393 (MRSA) and *Enterococcus faecalis* ATCC 29212/*Enterococcus faecium* clinical isolates, respectively (highlighted in red). Positive and solvent controls were also included (to show no bacterial growth and the target growth when exposed only to the extract solvent DMSO 20% v/v*,* respectively)

In this study, mild to potent antimicrobial activities were observed with *Planctomycetota* from all the nine genera tested, as represented in Fig. 2 (data available in Supplementary Table 1). Regarding the anti-fungal screenings, both *T. rubrum* FF5 and *C. albicans* ATCC 10231 showed susceptibility towards several planctomycetal extracts (Fig. 2). Additionally, extracts from strains *Rhodopirellula aestuarii* ICT_H3.1^ T^, *Rhodopirellula lusitana* LzF4 and *Novipirellula caenicola* MEMO17_8 induced a clear visual modification on the growth of *A. fumigatus* ATCC 240305 (such as the inhibition of the production of hyphae or abnormal morphology). Regarding the antibacterial screening, extracts of seven planctomycetal strains consistently inhibited the growth of methicillin-sensitive *S. aureus* ATCC 29213 in the three assays (growth reduction above 95%): *Rubinisphaera margarita* ICM_H10^T^, *Rubinisphaera brasiliensis* Gr7, *Roseimaritima ulvae* UC8^T^, *Rhodopirellula aestuarii* ICT_H3.1^ T^, *Aeoliella straminimaris* ICT_H3.7, *Rhodopirellula baltica* ICT_H6.1 and *Novipirellula caenicola* MEMO17_8. Partial activities (> 50% growth inhibition) were also observed with other planctomycetal strains (Fig. 2). On the other hand, only low activities were detected against *E. coli* ATCC 25922, where the only significant activity (> 50%) was observed with the extract of *Roseimaritima ulvae* UC8^T^ (52%). These results suggest an overall activity which is more specific towards Gram-positive bacteria. Gram-positive bacteria have a cell wall composed only of peptidoglycan and Gram-negative bacteria have an extra outer membrane. This difference may justify the greatest inhibition against Gram-positive bacteria because the bioactive molecules may interfere with peptidoglycan synthesis and/or may be unable to enter the cell due to the outer membrane barrier. Based on this finding, the six most bioactive strains were additionally screened against five Gram-positive pathogens, including antibiotic resistant strains. As summarized in Table 2 and in Supplementary Table 2, three *Planctomycetota* (*Rubinisphaera margarita* ICM_H10^T^, *Rhodopirellula aestuarii* ICT_H3.1^ T^ and *Rhodopirellula lusitana* LzF4) induced moderate to strong inhibitory effects across all the different pathogens tested, which is a strong indication for the production of bioactive molecules by these strains. Particularly, the extract of *R. margarita* ICM_H10^T^ inhibited the growth of all *Enterococcus* spp. tested, including vancomycin resistant ones (VanA and VanB, being VanA the most resistant), as it is depicted in Fig. 3. Additionally, *Novipirellula caenicola* MEMO17_8 showed a strong inhibitory effect on the methicillin-resistant *S. aureus* (MRSA) while affecting none of the *Enterococcus* spp. strains (Fig. 3).

**Table 2:** Heat map of the inhibitory effects of six planctomycetal extracts against a panel of five Gram-positive pathogens

Planctomycetal strain	Gram-positive target				
	^1^MRSA	^2^*E. faecalis*	^3^*E. faecium* VanS	^3^*E. faecium* VanB	^3^*E. faecium* VanA
ICT_E10.1^ T^	Low	No	No	No	No
ICM_H10^T^	Moderate	Potent	Potent	Potent	Potent
ICT_H3.7	Moderate	Low	Low	Low	Low
ICT_H3.1^ T^	Moderate	Moderate	Potent	Moderate	Moderate
LzF4	Moderate	Moderate	Potent	Moderate	Moderate
MEMO17_8	Potent	No	No	No	No

No activity: < 30% growth inhibition, low activity: 30–50% growth inhibition, moderate activity: 50–70% growth inhibition, potent activity: > 70% growth inhibition

^1^Methicillin-resistant *Staphylococcus aureus* MB 5393

^2^*Enterococcus faecalis* ATCC 29212

^3^Clinical isolates of *Enterococcus faecium*

In summary, diverse planctomycetal strains from different taxonomic groups within three families (*Lacipirellulaceae*, *Pirellulaceae* and *Planctomycetaceae*) from class *Planctomycetia* showed promising antimicrobial activities. Highest activities were observed against *T. rubrum* and methicillin-sensitive *S. aureus* (Fig. 2 and Supplementary Fig. 1). The strains with the widest range of activities towards the tested targets belong to the genera *Rhodopirellula* (*Rhodopirellula aestuarii* ICT_H3.1^ T^ and *Rhodopirellula lusitana* LzF4) and *Novipirellula* (*Novipirellula caenicola* MEMO17_8). *Rubinisphaera margarita* ICM_H10^T^ also stood out by having a broad effect across all tested Gram-positive bacteria.

With this study, we widen the spectrum of studied planctomycetal species for antimicrobial purposes. Our results reinforce the idea that many *Planctomycetota* (specifically, those of the class *Planctomycetia*) can indeed produce antimicrobial metabolites, most likely related to survival in their complex lifestyle and habitats such as biofilms, which has been also evidenced previously by genome mining (Belova, et al. 2020; Graca, et al. 2016; Jeske, et al. 2013; Kallscheuer and Jogler 2021; Wiegand, et al. 2020).

### Cytotoxic effects of planctomycetal extracts

**Fig. 4:**
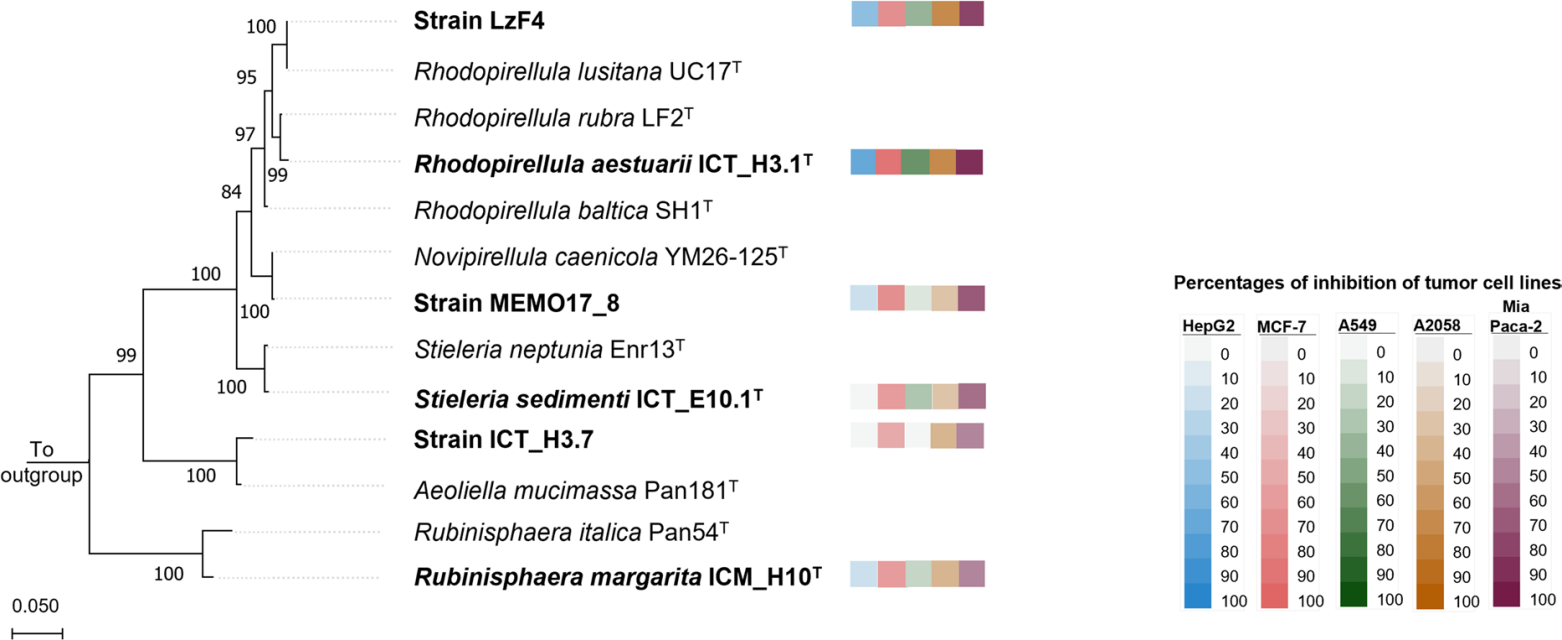
Phylogenetic representation of the planctomycetal strains screened for anti-tumor activities in this study (in bold) together with a heat map showing the effects against each cell line: HepG2 = human hepatocellular carcinoma; MCF7 = human breast cancer; A549 = human lung carcinoma; A2058 = human melanoma and MiaPaca-2 = human pancreatic cancer. Data used for construction of this tree is presented in Supplementary Table 3

Extracts from six planctomycetal strains that showed potent antimicrobial activities were additionally screened for cytotoxic effects against various cancer cell lines (data available at Supplementary Table 3). As represented in the phylogenetic tree combined with the heat map of the results obtained (Fig. 4), all extracts tested exhibited mild to potent activity against, at least, one cancer cell line. Overall, higher activities were observed towards human breast (MCF-7), pancreas (MiaPaca-2) and melanoma (A2058) cell lines (Fig. 4). Extracts from strains of the genus *Rhodopirellula* demonstrated the strongest effects against most cell lines. Extracts from the other 4 strains exhibited more selective activities to specific cell lines without affecting the most sensible one (HepG2), which is often used as a control for cytotoxicity.

Up to date, little is still known about the anti-cancer potential of the bacteria from the phylum *Planctomycetota*. Only one previous *in-vitro* screening study has identified promising strains and demonstrated the cytotoxic effects of various planctomycetal species against human prostatic cancer cell line PC3 and human acute myeloid leukemia cell line MOLM-13, as well as normal rat kidney epithelial cell line (Calisto, et al. 2019). MOLM-13 was the most susceptible cell line to planctomycetal extracts. Overall, *Rhodopirellula* spp. were the most cytotoxic, similarly to what we also observed in the present research. Our study contributes to broadening the number of planctomycetal species screened for anti-tumour activities and has identified promising strains from the species *Novipirellula caenicola*, *Stieleria sedimenti*, *Rubinisphaera margarita* and *Aeoliella straminimaris*.

### Dereplication of bioactive extracts

**Fig. 5:**
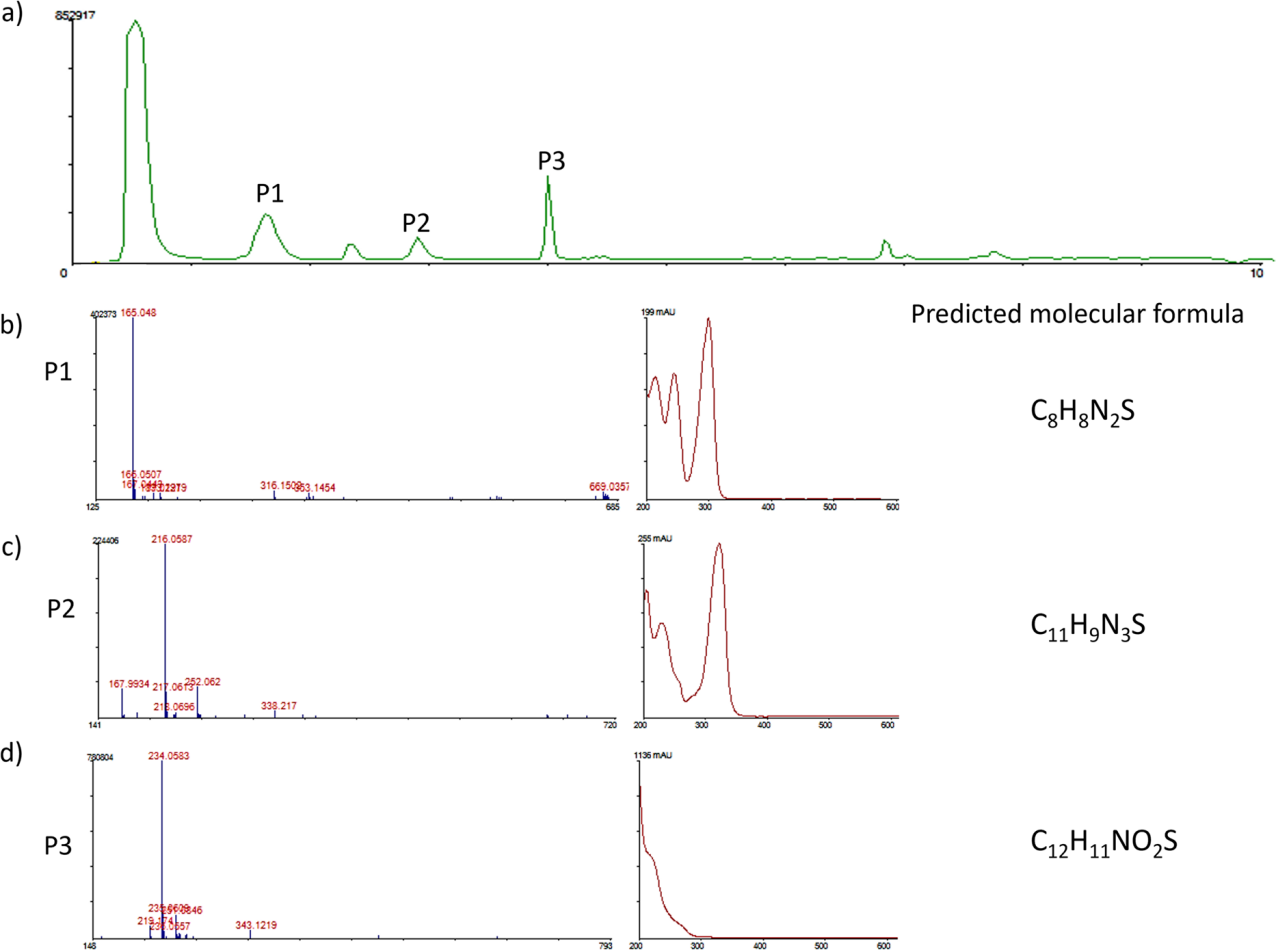
Chromatogram run (UV_210nm_) of the bioactive extract of strain MEMO17_8 (**a**) with 3 possible peaks of interest (P1, P2, P3), corresponding to putatively non-identified molecules. In the following order, the mass spectra and UV/vis of the components of Peak 1 (**b**), Peak 2 (**c**) and Peak 3 (**d**) are displayed, together with the molecular formulas suggested for each element

The chemical composition of some bioactive extracts was analysed through LC-HRMS. After searching for the exact mass or proposed molecular formula and UV/vis spectra in the DNP database of the components obtained, we could not identify any of the peaks with currently recognized bioactive natural products, therefore, the compounds responsible for the bioactivities observed remain unidentified (Fig. 5 and Supplementary Table 4). At this fermentation scale, biomass production by the planctomycetal strains is rather low, which leads to a low apparent complexity in the extracts. Nevertheless, several peaks were detected in the chromatographic runs, some corresponding to putatively non-identified molecules. For example, in the chromatogram run of *Novipirellula caenicola* MEMO17_8 bioactive extract (Fig. 5a), three major peaks were detected and their respective UV and mass spectra are also displayed (Fig. 5b, c and d). For each of these components, the predicted molecular formulae were the following: C_8_H_8_N_2_S for peak 1, C_11_H_9_N_3_S for peak 2 and C_12_H_11_NO_2_S for peak 3. While the first 2 were not found in the DNP, the molecular formula C_12_H_11_NO_2_S had a match for the compound named chuanghsinmycin, an antibiotic first isolated from a bacterium of the genus *Actinoplanes* (Shi, et al. 2018). However, the UV/vis spectra of both (peaks for Chuanghsinmycin at UV_228_, UV_285_ and UV_295_, according to literature) do not match, which seems to indicate that they might be distinct molecules.

Due to the phylogenetic distance of *Planctomycetota* with well-known prokaryotic producers of bioactive compounds (such as *Actinomycetota*), it can be hypothesized that their metabolomes (which are still largely underexplored) could have very distinctive profiles. This is also supported by genome analyses, which demonstrate that the phylum *Planctomycetota* currently contains a large number of unknown coding areas (thus coding for still unidentified compounds) (Faria, et al. 2018; Wiegand, et al. 2020, 2018). Additionally, when analysed for their content in BGCs and compared with known databases (for example, using AntiSMASH Blin, et al. 2021, 2019)), planctomycetal genomes rarely present strong identities to known gene clusters. All of this supports the likely production of novel metabolites by *Planctomycetota*, which is also reinforced by our chemical analysis.

### Oligotrophy as a means to obtain bioactive extracts from *Planctomycetota*

Secondary metabolites of bacterial origin are often bioactive molecules (Donadio, et al. 2007; Seyedsayamdost 2019), which implies that they are not always produced throughout all life cycle nor under all conditions. This implies that changes applied during bacterial fermentation can have a great impact on the molecules produced by each strain through deactivation/activation of metabolic pathways. Besides, it has also been perceived that even one strain can produce different compounds under distinct environmental conditions (the “One Strain, Many Compounds”- OSMAC approach) (Romano, et al. 2018; Santos, et al. 2022; Wei, et al. 2010). The same seems to be applicable for *Planctomycetota*. In previous planctomycetal studies, modifications in the content of the culturing medium changed the bioactivity outcome (Belova, et al. 2020; Graca, et al. 2016; Jeske, et al. 2016). Furthermore, Jeske and colleagues even obtained different molecule profiles when growing *Planctomycetota* in a maintenance medium supplemented with two different organic sources, in this case, glucose or *N*-acetylglucosamine (Jeske, et al. 2016).

*Stieleria sedimenti* strain ICT_E10.1^ T^ showed promising enhanced antimicrobial activity during oligotrophic stress (Vitorino, et al. 2022b). Thus, in this study, we also tried to apply stress through fermentation under oligotrophic conditions (medium 1:10 M13). The production of biomass under these conditions was greatly reduced when compared to the typical maintenance media such as M13 or M14 (Lage and Bondoso 2011). Yet, this condition seemed to be favourable for production of bioactive compounds, as many strains showed potent bioactivities. Already, a few of these strains have been screened for antimicrobial activities in previous studies against other groups of targets (Graca, et al. 2016; Vitorino, et al. 2022d). This is the case of strains Gr7, UC8^T^, LF2^T^, FF15^T^, Pd1, which were screened for activities against *Bacillus subtilis* and *C. albicans* (Graca, et al. 2016) and strains ICT_H3.1^ T^, ICT_E10.1^ T^, ICT_H6.1 and ICT_E8.1, which were screened against *E. coli*/*S. aureus* (Vitorino, et al. 2022d). In the first study, only strain Gr7 displayed mild inhibitory action towards both targets, while no activity was demonstrated by the other strains (Graca, et al. 2016). Strains ICT_H3.1^ T^, ICT_E10.1^ T^, ICT_H6.1 and ICT_E8.1 all demonstrated mild effects against the Gram-positive microorganism (Vitorino, et al. 2022d). However, the media used for fermentation was richer in comparison to the present study which used 1:10 M13 [M13/M14 and M14 supplemented with *N*-acetylglucosamine, respectively (Graca, et al. 2016; Vitorino, et al. 2022d)]. Since the fermentation of these same strains in oligotrophic medium either yielded new activities or enhanced previously observed ones, it is reinforced the need to test various growth conditions for the obtainment of planctomycetal bioactive extracts and the selection of promising strains.

## Conclusions

In this study, the majority of the 23 tested planctomycetal strains exhibited moderate to potent antimicrobial activities against selected pathogens. The six most potent planctomycetal strains also demonstrated mild to high effects against several human cancer cell lines. This study reinforces the relevance of *Planctomycetota* for biotechnological purposes. We also showed that more research is necessary to find out under which conditions the best bioactive extracts from *Planctomycetota* can be obtained. We have observed at least 3 putative novel compounds that need further molecular identification and testing.

## Supplementary Information

Below is the link to the electronic supplementary material.

Supplementary file1 (PDF 400 KB)

## Abbreviations

BGCs: Biosynthetic gene clusters
PKS: Polyketide synthases
NRPS: Non-ribosomal peptide synthetases
RiPP: Ribosomally synthesized and post-translationally modified peptide
rpm: Rotations per minute
DMSO: Dimethyl sulfoxide
CFU: Colony forming units
OD: Optical density
nm: Nanometers
LC/HRMS: Liquid chromatography/high-resolution mass spectroscopy
LEMUP: Laboratory for Microbial Ecophysiology of the University of Porto, Portugal
MRSA: Methicillin-resistant *Staphylococcus aureus*
VRE: Vancomycin-resistant *Enterococcus*
SDA: Sabouraud dextrose agar
