# Supplementary material for: Uncovering the biotechnological capacity of marine and brackish water Planctomycetota
Source: Antonie Van Leeuwenhoek. 2024 Jan 23;117(1):26. doi: 10.1007/s10482-023-01923-z (PMC10805854; doi:10.1007/s10482-023-01923-z)
Supplement: Supplementary file 1 — Supplementary file1 (PDF 400 KB) [file 10482_2023_1923_MOESM1_ESM.pdf]

Uncovering the biotechnological capacity of marine and brackish water *Planctomycetota*

Inês R. Vitorino[a,b], Eugénia Pinto[b,c], Jesús Martín[d], Thomas A. Mackenzie[d], Maria C. Ramos[d], Pilar Sánchez[d], Mercedes de la Cruz[d], Francisca Vicente[d], Vítor Vasconcelos[a,b], Fernando Reyes[d] and Olga M. Lage[a,b]

[a] Department of Biology, Faculty of Sciences, University of Porto, Rua do Campo Alegre s/n, 4169-007 Porto, Portugal

[b] CIIMAR/CIMAR, Interdisciplinary Centre of Marine and Environmental Research, Terminal de Cruzeiros do Porto de Leixões, 4450-208 Matosinhos, Portugal

[c] Laboratory of Microbiology, Biological Sciences Department, Faculty of Pharmacy, University of Porto, 4050-313 Porto, Portugal

[d] Fundación MEDINA, Avenida del Conocimiento 34, PTS Health Sciences Technology Park, 18016 Granada, Spain

Corresponding author: Inês Rosado de Jesus Vitorino, ines.rjv@gmail.com; ORCID: 0000-0002-5316-4493

Supplementary information

Supplementary Table 1. Data used for the construction of the heat map regarding the antimicrobial effects of planctomycetal crude extracts (Fig. 2), considering the biological replicates assayed for each target (n=3). For fungal assays, effects were considered when visually observed at least in two replicates.

	E. coli **ATCC 25922**		***S. aureus*** **ATCC 29213**		***A. fumigatus*** **ATCC 240305**	***T. rubrum*** **FF5**	***C. albicans*** **ATCC 10231**
**Planctomycetal strain**	Mean % inhibition	σ	Mean % inhibition	σ	Effect on growth		
Gr7	23	8.0	100	0.2	No inhibition	No inhibition	Partial reduction
LzF4	14	5.8	23	1.3	High inhibition	Total inhibition	High inhibition
ICT_H6.2^{T}	8	0.6	25	9.4	No inhibition	No inhibition	No inhibition
ICT_E10.1^{T}	1*	8.3*	100*	0.1*	No inhibition	No inhibition	No inhibition
ICT_H3.1^{T}	-22	1.3	100	0.1	Modified growth	Total inhibition	High inhibition
ICT_H6.1	11	3.9	99	3.5	No inhibition	No inhibition	No inhibition
ICM_H10^{T}	22	8.2	97	3.8	No inhibition	High inhibition	No inhibition
MsF2	12	3.4	27	4.9	No inhibition	No inhibition	No inhibition
MEMO26_1	13	8.2	20	2.3	No inhibition	No inhibition	No inhibition
MTI7a	15	8.3	43	7.7	No inhibition	High inhibition	No inhibition
ICM_H5	10	2.8	61	11.2	No inhibition	High inhibition	No inhibition
LzC2^{T}	20	2.5	18	6.8	No inhibition	No inhibition	No inhibition
FF15^{T}	27	4.9	17	1.1	No inhibition	No inhibition	No inhibition
UC8^{T}	52	6.8	97	10.5	No inhibition	High inhibition	No inhibition
ICT_H3.7	43	7.0	97	7.3	No inhibition	High inhibition	No inhibition
ICT_E8.1	36	5.6	39	9.8	No inhibition	No inhibition	No inhibition
LzA1	41	5.8	50	9.1	No inhibition	High inhibition	No inhibition
LzC1	34	10.3	59	7.8	No inhibition	High inhibition	No inhibition
MEMO17_8	33	4.2	99	1.3	Modified growth	High inhibition	Total inhibition
MTI8c	35	8.9	41	9.0	No inhibition	No inhibition	No inhibition
LF2^{T}	25	11.0	32	5.2	No inhibition	Total inhibition	No inhibition
ICM_G4	23	13.8	24	10.9	No inhibition	Partial reduction	No inhibition
Pd1	NT	NT	NT	NT	No inhibition	Partial reduction	No inhibition
Solvent control	6	2.5	6	3.6	No inhibition	No inhibition	No inhibition
Positive control	99	0.9	100	11.1	Inhibition	Inhibition	Inhibition

NT- not tested
* data from previous study (Vitorino*, et al.* 2022)
σ= Standard deviation

Supplementary Table 2. Mean percentages of inhibition of Gram-positive pathogens when exposed to planctomycetal extracts, considering the number of biological replicates performed for each target (n=3)

	**Gram-positive target**				
**Planctomycetal strain**	[1a]**MRSA**	[2b]***E. faecalis***	[3c]***E. faecium*** **VanS**	[3d]***E. faecium*** **VanB**	[3e]***E. faecium*** **VanA**
**ICT_E10.1^{T}**	32	10	17	12	12
**ICM_H10^{T}**	69	84	100	98	94
**ICT_H3.7**	50	35	41	35	33
**ICT_H3.1^{T}**	54	63	95	69	65
**LzF4**	61	62	85	58	59
**MEMO17_8**	78	-4	19	6	5

[1] Methicillin-resistant *Staphylococcus aureus* MB 5393, [2]*Enterococcus faecalis* ATCC 29212, [3] clinical isolates
[a]RZ' factor = 0.92, [b]RZ' factor =0.93, [c]RZ' factor =0.93, [d]RZ' factor =0.95, [e]RZ' factor = 0.88

Supplementary Table 3. Data used for construction of the heat map regarding the anti-tumor effects of crude planctomycetal extracts (Fig. 4), according to the number of biological replicates performed for each condition (n=2).

	**Mean % of inhibition of the tumor cell lines**				
**Planctomycetal strain**	**HepG2 (liver)**	**MCF-7 (breast)**	**A549 (lung)**	**A2058 (melanoma)**	**MiaPaca-2 (pancreas)**
LzF4	51	73	39	62	80
ICT_E10.1^T	5	59	32	38	59
ICT_H3.1^T	77	95	67	76	92
ICM_H10^T	23	60	22	40	53
ICT_H3.7	2	49	7	31	52
MEMO 17_8	21	52	11	37	77

Supplementary Table 4. LC-HRMS analysis of planctomycetal bioactive extracts.

**Strain ID**	**Putatively detected components**	**Suggested accurate masses**	**Matches for molecular formula in the prokaryotic DNP**
LzF4	C_8H_8N2S; $C_{11}H_9N_3S$; $C_{12}H_{11}NO_2S$	164.0406; 215.0512; 233.0506	$C_{12}H_{11}NO_2S$: Chuanghsinmycin, a compound isolated from *Actinoplanes tsinanensis* CPCC 200056 and from *Streptomyces nigrifaciens* MK219 (match for molecular formula but not for the UV/vis spectra)
ICM_H10	C_8H_8N2S; $C_{11}H_9N_3S$; $C_{12}H_{11}NO_2S$	164.0406; 215.0512; 233.0506	
MEMO17_8	C_8H_8N2S; $C_{11}H_9N_3S$; $C_{12}H_{11}NO_2S$; $C_{25}H_{44}O_3S_2$	164.0406; 215.0512; 233.0506; 456.2730	
ICT_H3.1	C_8H_8N2S; $C_{12}H_{11}NO_2S$	164.0406; 233.0506	
ICT_E10.1	C_8H_8N2S;$C_{12}H_{11}NO_2S$	164.0406; 233.0506	
ICT_H3.7	C_8H_8N2S; $C_{11}H_9N_3S$; $C_{12}H_{11}NO_2S$	164.0406; 215.0512; 233.0506	

Supplementary Figures

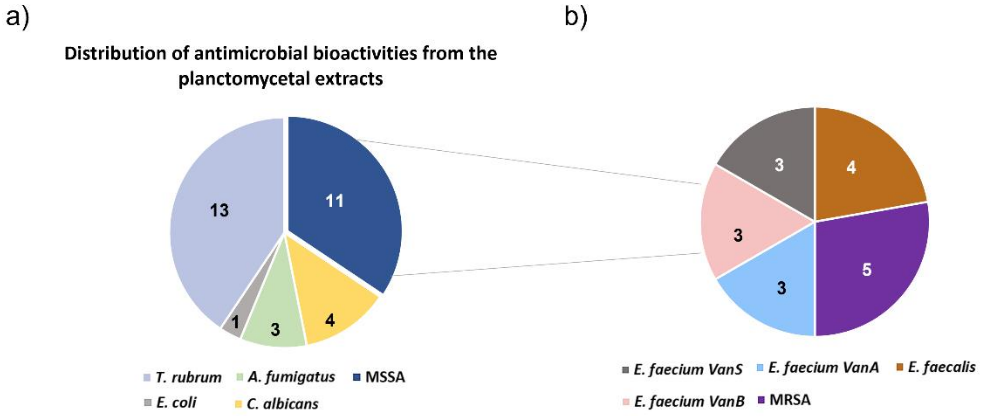

Supplementary Fig. 1 Distribution of the planctomycetal bioactivities (> 50% growth inhibition) across the microbial pathogens tested. Out of the twenty-three planctomycetes initially screened (a), thirteen strains displayed bioactivity against the fungi *Trichophyton rubrum* FF5, followed by eleven against methicillin-sensitive *Staphylococcus aureus* ATCC 29213. A small number of strains showed effects against *Candida albicans* ATCC 10231 and *Aspergillus fumigatus* ATCC 240305 while only one strain was bioactive against *Escherichia coli* ATCC 25922. Out of the six planctomycetes screened for a wider panel of Gram-positive bacteria (b), five strains were bioactive against methicillin-resistant *Staphylococcus aureus* MB 5393 (MRSA), four against *Enterococcus faecalis* ATCC 29212 and three against the various *Enterococcus faecium* clinical isolates
